# The relationships between subclinical OCD symptoms, beta/gamma-band power, and the rate of evidence integration during perceptual decision making

**DOI:** 10.1016/j.nicl.2022.102975

**Published:** 2022-02-28

**Authors:** Alec Solway, Isabella Schneider, Yuqing Lei

**Affiliations:** a Department of Psychology, University of Maryland-College Park, United States; b Program in Neuroscience and Cognitive Science, University of Maryland-College Park, United States

**Keywords:** Beta, Gamma, Decision making, Obsessive-compulsive disorder, Computational modeling, Drift-diffusion model

## Abstract

Previous studies have demonstrated that the rate of evidence integration during perceptual decision making, a specific computationally defined parameter, is negatively correlated with both subclinical symptoms of OCD measured on a continuum and categorically diagnosed patient status. However, the neural mechanisms underlying this deficit are unknown. Separate work has shown that both gamma and beta-band power are related to evidence integration, and differences in beta-band power in particular have been hypothesized to hinder flexible behavioral control. We sought to unify these two disparate literatures, one on OCD-related information processing differences constrained by behavioral data alone, and the other on the neural correlates of evidence integration. Using computational modeling and scalp EEG, we tested (N = 67) the relationships between subclinical symptom scores, drift rate, and gamma/beta-band activity during perceptual decision making. We replicated both prior work showing deficits in evidence integration as a function of OCD symptoms, and work showing a relationship between evidence integration and gamma and beta-band power. As predicted, the slope of beta-band power was correlated with OCD symptoms. However, the relationships between OCD symptoms and drift rate and the slopes of gamma and beta-band power and drift rate remained unchanged when simultaneously accounting for all variables, speaking against the hypothesis that differences in band-band power explain drift rate deficits.

## Highlights

• Drift rate previously shown to be disrupted in both clinical and subclinical OCD.
• Separately, evidence integration has been linked to gamma and beta-band power.
• Tested whether the slope of beta-band power mediated drift rate/OCD relationship.
• As expected, slope of beta-band power was related to subclinical symptoms.
• However, it did not explain the relationship with drift rate.

## 1 Introduction

A number of previous studies have identified a specific computationally defined deficit in perceptual decision making that is associated with both subclinical symptoms of OCD measured on a continuum and categorical patient status (Banca et al., 2015, Erhan et al., 2017, Hauser et al., 2017, Marton et al., 2019). These studies made use of the dot motion task and the drift-diffusion model. In the dot motion task, subjects view moving dots and are asked to determine in which direction (e.g. left/right) the dots are displaced on average (Newsome et al., 1989, Ratcliff and McKoon, 2008). Difficulty can be varied by changing the level of coherence—the proportion of dots moving in unison. Information processing in this task is well-explained by the drift-diffusion model, which provides a unifying account of how decisions between two options evolve within multiple domains and tasks (Mormann et al., 2010, Ratcliff, 1978, Ratcliff and McKoon, 2008). In the model, the relative amount of evidence for one option versus the other undergoes a noisy trajectory during deliberation, with its mean velocity represented by a free parameter called *drift rate*. A decision is made when the relative amount of evidence reaches one of two decision boundaries, representing the options under consideration. A smaller distance between the boundaries indicates the subject requires less evidence to reach a decision, and vice versa. A pre-existing preference for one of the options, before seeing the stimulus, is represented by a concordant change in the amount of relative evidence at the start of the decision. Finally, a fourth free parameter codes the time taken for non-decision aspects of each trial. The studies noted above showed that OCD symptoms were negatively correlated with drift rate, the average amount of perceptual evidence integrated per unit time, in the dot motion decision task. Furthermore, these impairments were larger for easier (higher coherence) decisions, and were absent for the hardest decisions (Banca et al., 2015, Marton et al., 2019). In contrast, perhaps surprisingly, the majority of these studies did not find that OCD symptoms were related to less impulsive and more deliberate decision making (i.e. wider decision boundaries).

Although the drift rate finding has been replicated multiple times by independent groups, the neural correlates of this deficit have not been studied. The use of the drift-diffusion model in this context, in addition to providing a formal and explicit description of decision making, allows us to link this developing literature to the larger literature on basic decision neuroscience. In the case of the dot motion task, a number of studies have shown that moment-by-moment decision evidence is represented by area MT, and is passed to downstream areas in frontal and parietal cortex to be integrated in a fashion similar to that described by the drift-diffusion model (Gold and Shadlen, 2000, Gold and Shadlen, 2007, Heekeren et al., 2004, Huk and Shadlen, 2005). Studies using non-invasive recording techniques in humans, although suffering from poorer spatial resolution, have also uncovered neural signatures of evidence integration using more varied perceptual tasks, helping link specific aspects of the model and the model’s parameters to neural data (Donner et al., 2009, Polanía et al., 2014, Philiastides et al., 2014). MEG (magnetoencephalography) and EEG (electroencephalography) are of particular interest here because although their spatial resolution is poor, their temporal resolution allows for the tracking of evidence integration at the timescale at which it unfolds.

Studies focusing on spectral properties have found consistent evidence that both gamma and beta-band power is correlated with evidence integration ((Donner et al., 2009, Polanía et al., 2014); though see also (van Vugt et al., 2012)). Donner et al. (2009) asked participants to perform a variant of the dot motion task in which the decisions were about the absence or presence of a signal (trials with 0% or positive coherence) while recording MEG activity. They found that: gamma-band (64–100 Hz) power in premotor and motor cortex ramped up during decision making in a fashion analogous to the process described by the drift-diffusion model, that it could be used to predict decisions before a response was issued, and that motor cortex activity reflected the integral of gamma-band power in area MT. Similarly, Polanía et al. (2014) asked participants to perform both a perceptual task in which they made a judgment about which stimulus covered a larger area of the screen, and a reward-based task where they decided which of two foods they would rather have. EEG-measured gamma-band power (46–66 Hz) ramped up during the course of both perceptual and reward-based decision making as predicted by an evidence integration model very similar to the drift-diffusion model. In addition, both studies found that beta-band power *negatively* correlated with evidence integration (12–36 Hz in Donner et al. (2009) and 18–20 Hz in Polanía et al. (2014), although this was true only for the reward-based task in the latter study).

Given the relationships between changes in gamma and beta-band power and evidence integration, the *slope* of power in each band is a candidate mediator of the previously reported association between OCD symptoms and drift rate. While previous work directly connecting power in each band to OCD symptoms is more limited, beta-band power has been suggested to reflect the maintenance of the current cognitive state or ‘status quo’ (Engel and Fries, 2010). The same authors further hypothesized that abnormally high levels of beta-band power would hinder flexible behavioral control and be associated with OCD symptoms. While their prediction was not about slope per se but about absolute levels of beta-band power, in the present context using the same reasoning we might expect that a smaller change (a less negative slope) would be associated with OCD symptoms. Combining all of the above—the negative correlation between drift rate and OCD symptoms, the relationships between changes in gamma and beta-band power and evidence integration, and the hypothesis that differences in beta-band power in particular may be related to OCD symptoms—the present work aimed to test whether beta (and for completeness gamma) band power was a neural mediator of the drift rate/OCD relationship. We note however that these are not the only candidate M/EEG-based mediators. For example, the centro-parietal positivity has also been shown to be related to evidence integration and specifically drift rate ((O’Connell et al., 2012, van Vugt et al., 2019); see also (Philiastides et al., 2006, Philiastides et al., 2014)), and our focus on particular spectral features in the current work is not meant to suggest that other EEG features should be ruled out a priori.

Participants were recruited based on a dimensional approach to measuring psychopathology, covering a wide range of symptom severity from the low to the high-end (Burns et al., 1996). Symptoms of worry (Meyer et al., 1990), a primary characteristic of generalized anxiety disorder (American Psychiatric Association, 2013), were also measured to test the specificity of the results. Participants performed the standard dot motion task in tandem with EEG data recording. We asked first whether we could again replicate the negative relationship between OCD symptoms and drift rate, and whether these deficits were larger for easier decisions. Second, we tested whether the dynamics of beta and gamma-band power during deliberation were related to evidence integration. We did this in two ways: 1) we examined whether power in each band gradually changed during the course of decision making (ramping up or down) and whether ramps were steeper during easier decisions, and 2) in addition, across subjects, we asked whether the slope of evolving beta and gamma-band power was related to drift rate measured using behavioral data alone. Third, we tested the relationship between OCD score and the slope of power in each band. Finally, we tested whether the relationship between OCD score and drift rate was reduced when also accounting for beta and gamma-band power.

## 2 Methods

**Participants.** All experimental procedures were approved by the Institutional Review Board at the University of Maryland-College Park. Informed consent was obtained in accordance with the approved procedures. Participants were drawn from the campus and the surrounding community. In all, 67 participants took part in the study. Potential participants were pre-screened in attempt to evenly cover a range of Padua Inventory scores (Burns et al., 1996), with the following bin boundaries: 0–16, 17–33, 34–50, 51+. The Padua Inventory is a standard instrument also used in prior work looking at the relationship between dimensional OCD symptom scores and perceptual deficits (Hauser et al., 2017). While we advocate a dimensional approach, for reference previous work has shown patients scoring 55 on average, versus 22 for controls (Burns et al., 1996). Participants also completed the Padua Inventory on the day of the experiment. SI Fig.S1 displays the relationship between pre-screen and same day scores. Same day scores were used in all analyses. Despite some changes between the two testing sessions (as expected), we had a good balance among the a priori defined bins, with 19, 18, 15, and 15 participants respectively in the four groups. Bins were used for pre-screening to ensure coverage of a range of scores, but the Padua Inventory is a continuous measure and all analyses treated it as such. We tested whether results were specific to OCD symptoms by controlling for and asking the same questions about symptoms of worry measured using the Penn State Worry Questionnaire collected on the day of the experiment (Meyer et al., 1990). While as above we advocate a dimensional approach, for reference prior work has suggested cutoff scores of 62–65 for diagnosing generalized anxiety disorder using this measure (Behar et al., 2003, Fresco et al., 2003). SI Fig.S2 plots the distribution of scores from both questionnaires. There was evidence of some skew in the worry scores towards the upper range. Participants were not screened for categorically defined current or past disorders. Unfortunately, demographic information was lost for 20 participants. Of the remaining participants, 11 were male and 36 were female, with age ranging from 18–25 (one participant did not give their age). The restricted age range and unbalanced gender composition of the sample are limitations of the study.

**Dot motion task.** The dot motion task was programmed in Matlab (The MathWorks, Inc.) using Psychtoolbox (Brainard, 1997), based on publicly available code from Michael Shadlen’s laboratory (Shadlen Lab, 2018). We utilized six different coherence (difficulty) levels based on a previous study looking at perceptual deficits in OCD (Banca et al., 2015): 0.025, 0.05, 0.15, 0.25, 0.45, and 0.7. On average, the corresponding percentage of dots moved coherently right or left (selected randomly on each trial) while the remaining dots appeared at random. In addition to specifying the direction of motion, participants simultaneously rated the confidence in their response as “Very Certain”, “Certain”, or “Somewhat Certain”. Participants gave a “left” response using the Q, W, and E keys (corresponding to the respective confidence categories), and a “right” response using the P, O, and I keys. Participants completed a demo and practiced positioning their hands before starting the main portion of the experiment. There were 120 trials for each coherence level randomly intermixed throughout the experiment. A break was offered every 120 trials. For analysis, trials with reaction times faster than 250 ms and slower than 10s were removed. This resulted in 3 out of 402 participant/condition combinations having less than 100 valid trials (53, 77, and 90, all associated with the two hardest conditions); all other participant/condition combinations had 100 or more valid trials.

**EEG methods.** EEG data were recorded using actiCAP electrode caps, the actiCHamp amplifier, and Pycorder recording software, all from Brain Products. We recorded 64 channels, with a 500 Hz sampling rate, using electrode FCz as the reference. Data were analyzed using FieldTrip (Oostenveld et al., 2011) in Matlab. During pre-processing, data were re-referenced to the average of channels T7 and T8. Eye-blinks were removed using independent component analysis. The scalp current density was computed using the *spline* method implemented in the ft_scalpcurrentdensity function. Time-frequency analysis was performed using multitapers (*mtmconvol* method in ft_freqanalysis, with the default *dpss* for the taper type). For gamma, frequencies of interest were 32–100 in increments of 4 Hz. For beta, frequencies of interest were 12–28, also in increments of 4 Hz. A 250 ms time window and ±8Hz of smoothing was used. Power was log-transformed and normalized on a per-subject/per-condition/per-channel/per-frequency basis by subtracting the mean and dividing by the standard deviation of power during the 200 ms period preceding each stimulus onset. Normalized power was then averaged across frequencies within each band.

**Whole-brain measure of evidence integration.** Based on prior work, we derived an aggregate whole-brain neural measure of evidence integration (Philiastides and Sajda, 2006, Philiastides et al., 2014), although some of the details have been modified. We used multi-level Bayesian logistic regression to predict trial difficulty (grouping 0.025 and 0.05 coherence trials as “hard”/0 and 0.45 and 0.7 trials as “easy”/1) from band-specific power at all electrode sites, using the median reaction time for hard trials as the time point of interest. We fit the model using Markov chain Monte Carlo implemented in rstanarm (Goodrich et al., 2020) v2.21.1, using the default weakly informative priors for the coefficients (centered and biased towards 0). In addition to the use of regularizing priors, this general framework allowed us to constrain the fits through partial pooling as in all standard multi-level models: Regression coefficients for participants were assumed to arise from a common group-level Gaussian distribution, thus constraining each other’s fit in a data driven manner (Gelman et al., 2013). We ran four chains and used 1,000 samples for warmup and 3,000 samples for inference for each chain. The R^ statistic was <1.05 for all variables. After fitting the model, the entire distribution of coefficients was used to weigh the data for each electrode, trial, and time point.

**Fig. 4:**
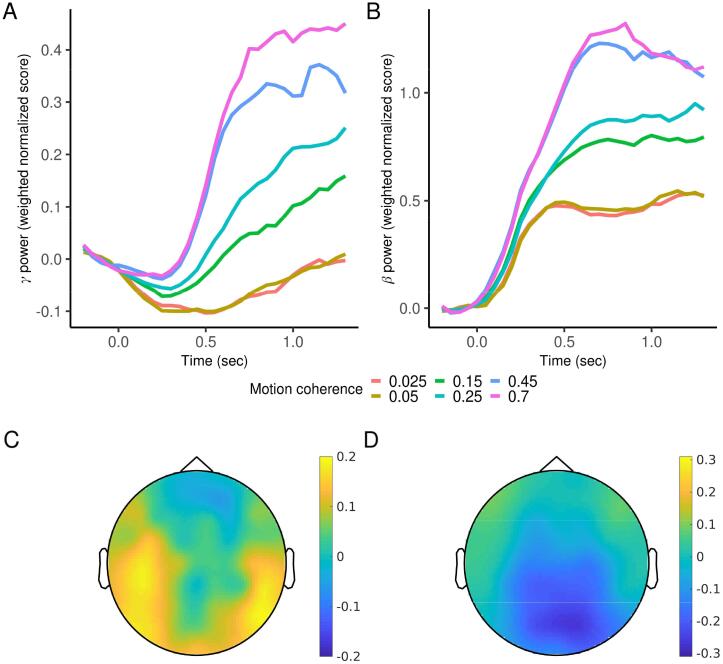
A-B. Mean aggregate whole brain gamma and beta-band power for each condition aligned to the start of the decision. The weight of each electrode was estimated using a multi-level regression predicting easy (0.45 and 0.7 coherence) vs hard (0.025 and 0.05) trials, based on power at the median reaction time for hard trials for each participant. The weights were then applied to the data for all trials and time points. Note that based on how it is defined using logistic regression, the aggregate measure is positive when the magnitude of power at individual electrodes is overall greater for easier decisions, regardless of whether the difference is positive or negative (and the aggregate measure would be negative if the magnitude was greater for harder decisions). C-D. Projection back to the scalp computed by correlating the aggregate measure with power at individual electrodes (C. γ power, D. β power).

**Fig. 5:**
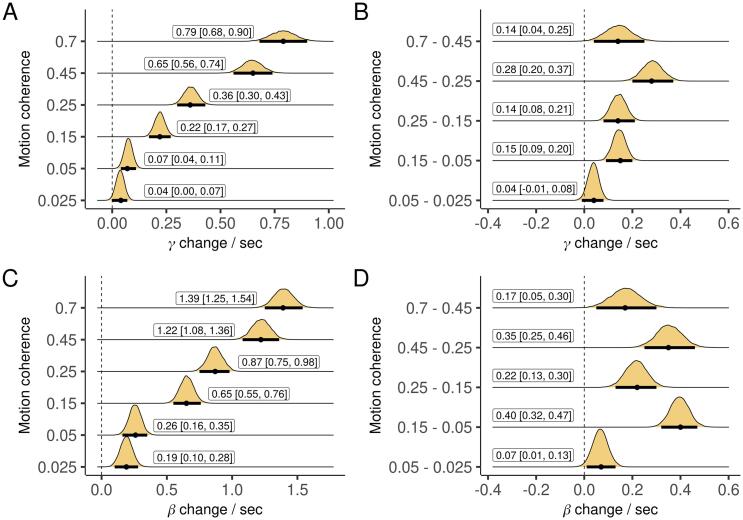
The slope of aggregate gamma and beta-band power estimated between 300 and 800 ms (see *Methods*) for each condition, and differences between conditions. Plots are marginal posterior distributions with medians and 95% credible intervals noted.

To compute the plots shown in Fig. 4, aggregate weighted power was averaged (sample by sample, for each sample from the Markov chain Monte Carlo procedure estimating the Bayesian logistic regression model) separately for each condition and time point first within subject across trials, and then across subjects. The medians of the resulting marginal distributions were used for display purposes. To compute the slope of the aggregate measure (Fig. 5), after averaging within subject for each condition, we computed differences in 50 ms increments spanning the 500 ms period from 300 to 800 ms, took their mean, and then the mean across subjects. For the analysis of individual differences (described below), the median of the marginal distribution for each subject was used. Note that the overall analysis is only approximately Bayesian. A fully Bayesian analysis would perform these computations together with the drift-diffusion and regression model fits within a single overarching model, although this proved computationally infeasible.

To project aggregate power back to the scalp, we first computed for each subject: *XY*, where X is a channels x time matrix of power at each individual channel and time point, and Y is a time x samples matrix representing aggregate power (Philiastides and Sajda, 2006, Philiastides et al., 2014). Each column (sample), *j*, in the resulting matrix was then normalized by $Y_{\cdot ,j}^{T}Y_{\cdot ,j}$. The mean was taken across subjects, and finally the median was extracted across samples for each channel.

**Drift-diffusion modeling.** A multi-level Bayesian framework implemented in Stan (Stan Development Team) was also used to fit the drift-diffusion model. We fit the simple form of the model without variance in parameters across trials. Although this version cannot explain differences in reaction time between correct and error trials (Ratcliff and McKoon, 2008), simulation work has shown that it can provide more robust parameter recovery than the full version, and is useful if the variance parameters are not of interest (Lerche and Voss, 2016). As above, we ran four chains and used 1,000 samples for warmup and 3,000 samples for inference in all analyses. The R^ statistic was ⩽1.05 for all variables. Broad uninformative priors were used at the group level. Priors are described below following the description of the regression models.

**Regression models.** Regression models were fit together with the drift-diffusion model in Stan. The final full regression was defined as follows for subject *s* and condition *c*:

(1) $$\begin{array}{cc} {\mathrm{drift}}_{s,c}= & {\mathrm{drift}}_{c}+{\mathrm{drift}}_{s}+\\ & {\mathrm{drift}}_{c,\mathrm{padua}}\cdot {\mathrm{padua}}_{s}+{\mathrm{drift}}_{c,\mathrm{worry}}\cdot {\mathrm{worry}}_{s}+\\ & {\mathrm{drift}}_{c,\gamma }\cdot \gamma_{s,c}+{\mathrm{drift}}_{c,\beta }\cdot \beta_{s,c}\text{.} \end{array}$$

${\mathrm{padua}}_{s}$ and ${\mathrm{worry}}_{s}$ are z-scored symptom scores, and $\gamma_{s,c}$ and $\beta_{s,c}$ are the subject and condition specific slopes of aggregate band-specific power, z-scored for each condition. The boundary was similarly defined. The two reduced sets of regressions had a similar form, one without the symptom regressors and one without the neural regressors. Another pair of regressions tested the relationship between the slope of aggregate power in each band and OCD and worry scores:

(2) $${\mathrm{neural}}_{s,c}=b_{c,0}+b_{c,\mathrm{padua}}\cdot {\mathrm{padua}}_{s}+b_{c,\mathrm{worry}}\cdot {\mathrm{worry}}_{s}+{∊}_{s,c},$$

where ${\mathrm{neural}}_{s,c}$ is one of $\gamma_{s,c}$ or $\beta_{s,c}$, and ${∊}_{s,c}$ is Gaussian noise.

**Priors for fitting the drift-diffusion and regression models.** The priors for all parameters were broad and uninformative. For the non-decision time parameter, the group-level mean had a N(0.5,1) prior (the second number in all such labels is SD), and the group-level standard deviation had a N(0,1) prior. The subject-specific non-decision parameters had a Gaussian prior with the aforementioned group-level parameters. The starting point parameter was fixed to 0.5 (halfway between the two options) for all analyses, as each of the 720 trials had a 50/50 probability of the underlying motion being either left- or rightward.

As noted above, regression models involving drift-diffusion model parameters were fit simultaneously with the drift-diffusion model. In order to reduce correlation in the sampled posterior, we sampled modified parameters ${\mathrm{drift}}_{c^{\prime }}$ where ${\mathrm{drift}}_{c=1}={\mathrm{drift}}_{c^{\prime }=1},{\mathrm{drift}}_{c=2}={\mathrm{drift}}_{c^{\prime }=1}+{\mathrm{drift}}_{c^{\prime }=2},{\mathrm{drift}}_{c=3}={\mathrm{drift}}_{c^{\prime }=1}+{\mathrm{drift}}_{c^{\prime }=3}$, and so on, and similarly for the drift rate regression coefficients and boundary separation parameters. All reported parameters are the original parameters (e.g. ${\mathrm{drift}}_{c}$). The priors for ${\mathrm{drift}}_{c^{\prime }},{\mathrm{drift}}_{c^{\prime },\mathrm{padua}},{\mathrm{drift}}_{c^{\prime },\mathrm{worry}}$, ${\mathrm{drift}}_{c^{\prime },\gamma },{\mathrm{drift}}_{c^{\prime },\beta },{\mathrm{boundary}}_{c^{\prime }\neq 1}$, ${\mathrm{boundary}}_{c^{\prime },\mathrm{padua}},{\mathrm{boundary}}_{c^{\prime },\mathrm{worry}},{\mathrm{boundary}}_{c^{\prime },\gamma }$, and ${\mathrm{boundary}}_{c^{\prime },\beta }$ were N(0,20). The prior for ${\mathrm{boundary}}_{c^{\prime }=1}$ was N(1,20), biased positive because the boundary separation must be positive, but with a large standard deviation that does not overwhelm the data. The priors for ${\mathrm{drift}}_{s}$ and ${\mathrm{boundary}}_{s}$ were hierarchically defined, $N(0,\sigma_{\mathrm{drift}})$ and $N(0,\sigma_{\mathrm{boundary}})$, with the priors for $\sigma_{\mathrm{drift}}$ and $\sigma_{\mathrm{boundary}}$ set to N(0,20).

The priors for $b_{c,\mathrm{padua}},b_{c,\mathrm{worry}}$, and the standard deviation of ${∊}_{s,c}$ were similarly N(0,20). The intercept $b_{c,0}$ was hierarchically defined, with the prior for $b_{c,0}$ set to $N(b_{\mu_{0}},b_{\sigma_{0}})$, and the priors for $b_{\mu_{0}}$ and $b_{\sigma_{0}}$ set to N(0,20).

## 3 Results

**Fig. 1:**
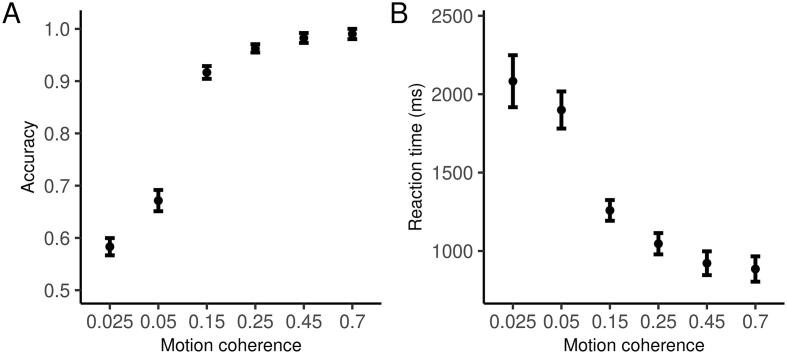
Accuracy and reaction time (for correct trials) for each difficulty condition.

**Basic behavioral results.** As a basic check of our experiment, Fig. 1 displays the mean accuracy and reaction time for each condition. Accuracy increased (F(5,330)=694.4, p<2e-16) and reaction time decreased (F(5,330)=178, p<2e-16, computed after taking the log due to the skew inherent to reaction time distributions) for easier (larger coherence) trials. Rather than focus on raw behavior, we modeled the data using the drift-diffusion model, which has been extensively studied and validated in the context of the dot motion task (Gold and Shadlen, 2007, Ratcliff and McKoon, 2008). This allowed us to analyze accuracy and reaction time in tandem, and ask about specific components of information processing. Model fitting was performed using a multi-level Bayesian framework. In the following, we plot the marginal posterior probability distributions of the parameters of interest, and report the central 95% credible intervals. As is common, we treat an effect as “significant” if there is more than a 95% probability that it is larger (or smaller depending on the prediction) than 0, although we encourage taking the entire posterior distribution and the uncertainty in measurement into account. All Bayesian statistical estimates are displayed in the figures rather than the text for compactness.

**Fig. 2:**
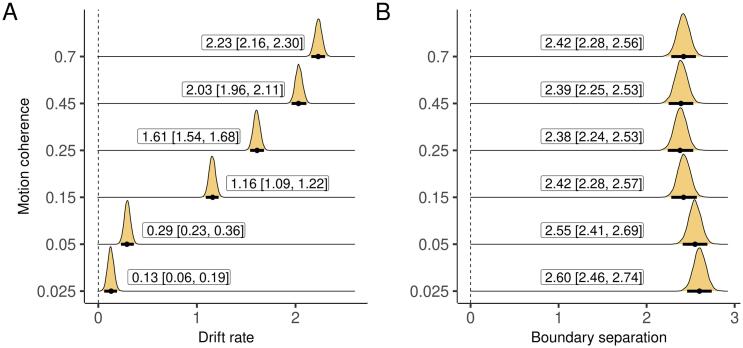
Estimated drift rate and boundary separation for each condition. Plots are marginal posterior distributions with medians and 95% credible intervals noted.

As expected, drift rate increased as a function of coherence (Fig. 2A; the difference between conditions is displayed in SI Fig.S3A). We also fit a separate boundary separation parameter for each condition. Note that although it is often assumed that boundary separation does not vary as a function of difficulty, and this constraint is enforced as part of the model fitting procedure, studies measuring OCD-related impairments that have allowed this parameter to vary have found differences across conditions (Banca et al., 2015, Marton et al., 2019). Consistent with these studies, there was some evidence that boundary separation was smaller for easier decisions, although this was not true for all conditions (Fig. 2B and SI Fig.S3B).

**Fig. 3:**
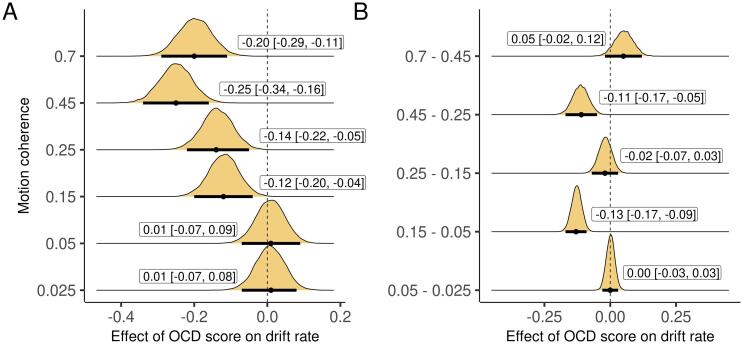
Effect of OCD score on drift rate, simultaneously controlling for worry, for each condition and the difference between conditions. Plots are marginal posterior distributions with medians and 95% credible intervals noted.

**Evidence integration was impaired as a function of OCD symptoms, but not worry.** Replicating prior work (Banca et al., 2015, Erhan et al., 2017, Hauser et al., 2017, Marton et al., 2019), OCD score was negatively associated with drift rate during easier decisions, with significantly larger effects for easier compared to harder conditions (Fig. 3). The displayed effects were computed while simultaneously controlling for worry, with the reduction in drift rate specific to OCD score. There was some evidence that drift rate was actually larger as a function of worry, although only in some conditions (SI Fig.S4A-B). Importantly, the differences between the effects of OCD score and worry on drift rate were significant for all conditions where OCD score was related to drift rate (SI Fig.S4C). Boundary separation did not vary either as a function of OCD score (SI Fig.S5) or worry (SI Fig.S6).

**Changes in both gamma and beta-band power were related to evidence integration.** Prior studies describing the relationship between gamma and beta-band power and evidence integration reported results in somewhat idiosyncratic frequencies (Donner et al., 2009, Polanía et al., 2014). We first sought to replicate this work using a broader range of frequencies within each band: 32–100 Hz for the gamma band and 12–28 Hz for the beta band (Engel and Fries, 2010, Gluth et al., 2013, Long et al., 2014). We used an approach from a complementary set of studies to calculate an aggregate whole-brain measure of power over time for each band of interest (Philiastides and Sajda, 2006, Philiastides et al., 2014), and this formed the basis for our main statistical questions. We also projected this measure back to sensor space to study its gross spatial distribution, as described below.

To calculate this measure, we computed average power within each respective range and used multi-level Bayesian logistic regression to predict trial difficulty (grouping 0.025 and 0.05 coherence trials as “hard”/0 and 0.45 and 0.7 trials as “easy”/1) from power at all electrode sites. This analysis was performed at the median reaction time for hard trials (see *Methods*). We then used the fitted regression coefficients to weigh each electrode and compute an aggregate measure of power for all conditions (including now the medium difficulty conditions) and all time points (not just the single time point corresponding to the median reaction time for hard trials). Note that because of how it is defined based on logistic regression, the aggregate measure is positive when the *magnitude* of power at individual electrodes is overall greater for easier decisions, regardless of whether the difference is positive or negative (and the aggregate measure would be negative if the magnitude was greater for harder decisions).

**Fig. 6:**
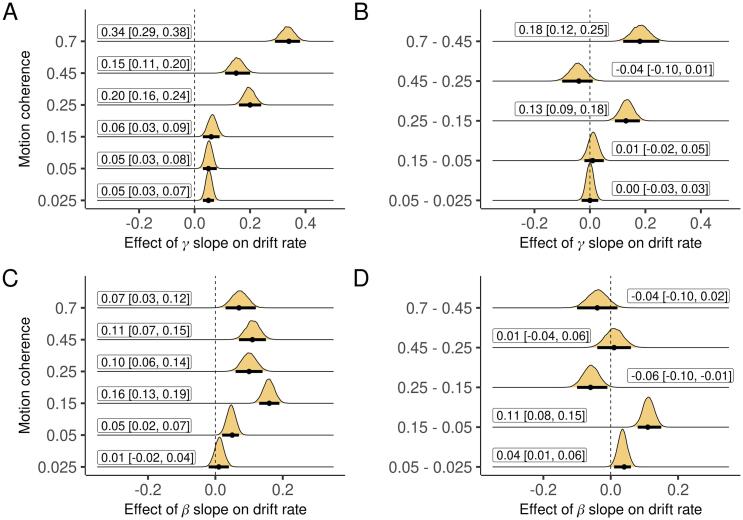
Effect of the slope of aggregate γ and β power on drift rate across subjects, each while controlling for the other, for each condition and differences between conditions. Plots are marginal posterior distributions with medians and 95% credible intervals noted.

Fig. 4A-B displays the mean of the aggregate measure separately for each band and condition as the decision unfolded. In line with previous EEG and MEG work (Donner et al., 2009, Polanía et al., 2014), and remarkably similar to the pattern seen in the firing rate of neurons in animal studies (Gold and Shadlen, 2007), the aggregate measure for both bands gradually increased during decision making. The rate of increase was higher for higher coherence (easier) trials (Fig. 5). We projected the aggregate measure back to sensor space by correlating it with power at each individual channel (Philiastides and Sajda, 2006, Philiastides et al., 2014). In addition to visualizing the spatial distribution of the measure, this analysis reveals the direction (positive or negative) of the ramps at each electrode. Gamma-band ramps were mostly positive and concentrated at posterior and lateral electrode sites, while beta-band ramps were mostly negative and concentrated at parieto-occipital electrode sites (Fig. 4C-D). There were also smaller effects in the opposite direction in each band at more frontal electrodes. Finally, we looked across participants, extracting the slope of the aggregate measure for each condition, participant, and band, and regressing drift rate on the slope of both bands simultaneously. Drift rate was independently correlated with the slope of the aggregate measure for gamma power in all conditions, and for beta power in five out of six conditions (Fig. 6A,C).

**Fig. 7:**
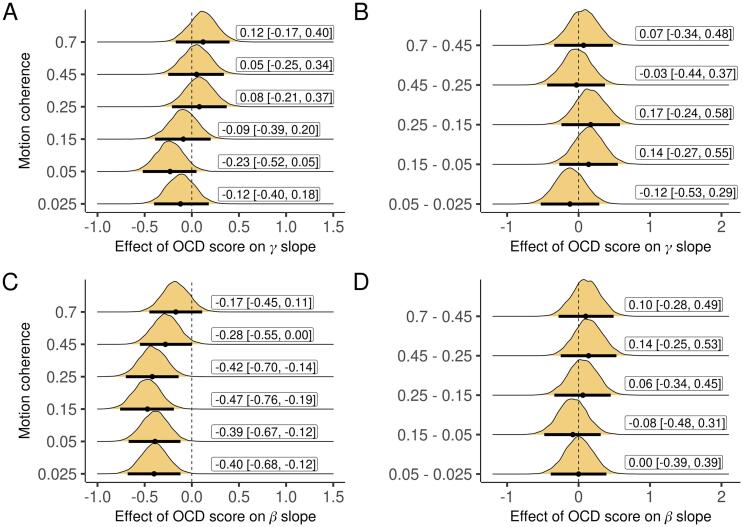
Effect of OCD score on the slope of aggregate γ and β power across subjects, controlling for worry, for each condition and the differences between conditions. Plots are marginal posterior distributions with medians and 95% credible intervals noted.

**Slope of beta but not gamma power was correlated with OCD score.** Next, we tested the relationship between the slope of aggregate power in each band and OCD score, again controlling for worry. The slope of gamma-band power was unrelated to both OCD (Fig. 7B) and worry (except a likely false positive in a single condition, SI Fig.S7A-B) scores. However, the slope of aggregate beta-band power was negatively correlated with OCD score in five out of six conditions (with a trend in the remaining condition; Fig. 7C). These effects were generally stronger than (non-significant) effects of worry (SI Fig.S7C,F).

**Fig. 8:**
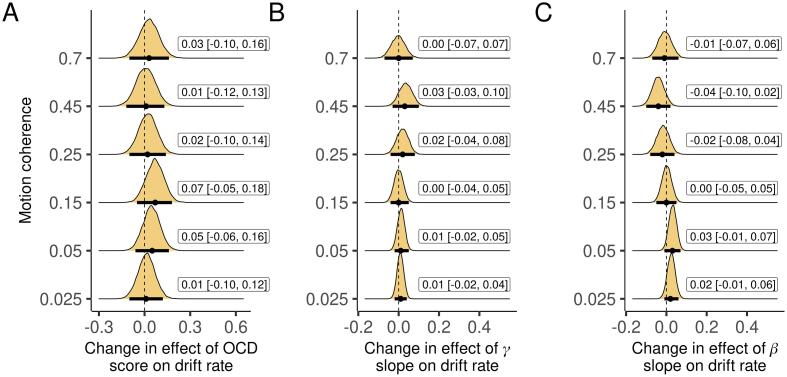
A. Change in the effect of OCD score on drift rate when controlling for aggregate γ and β slopes in addition to worry. B-C. Changes in the effects of aggregate γ and β slopes on drift rate when controlling for OCD score and worry. Plots are marginal posterior distributions with medians and 95% credible intervals noted.

**When simultaneously accounting for all variables, there was no reduction the effect of OCD score or the slope of aggregate power in either band on drift rate**. If the relationship between OCD score and the slope of aggregate beta-power accounts for the association between OCD score and drift rate, we should see a reduction in the effect of OCD score when drift rate is simultaneously regressed on both variables. We also controlled for the slope of aggregate gamma-band power and worry as in prior analyses. Neither the effect of OCD score (Fig. 8A) nor the slope of aggregate power in either band (Fig. 8B-C) significantly changed compared to the reduced models.

**Confounding from saccadic activity.** Saccades are known to elicit gamma-band responses, although computing the scalp current density attenuates this effect, at least at non-frontal electrodes (Keren et al., 2010). All of the described analyses were performed after computing the scalp current density (Perrin et al., 1989). In addition, we repeated all analyses after excluding the frontal electrodes (Fp1, Fp2, AF7, AF3, AFz, AF4, AF8, F7, F5, F3, F1, Fz, F2, F4, F6, F8). The results were qualitatively similar with these electrodes removed.

## 4 Discussion

We sought to synthesize the previously disparate literatures on the relationship between OCD symptoms and evidence integration (drift rate) during perceptual decision making, and the relationship between evidence integration and gamma and beta-band neural activity. Consistent with previous work, we found a negative relationship between OCD symptoms and drift rate, and this effect was larger for easier decisions (Banca et al., 2015, Erhan et al., 2017, Hauser et al., 2017, Marton et al., 2019). We did not find evidence of a relationship between decision boundary separation and OCD score. Although this may seem surprising, our findings are consistent with the literature, where only 2 out of 5 studies reported increases in boundary separation as a function of OCD (Banca et al., 2015, Erhan and Balci, 2017, Erhan et al., 2017, Hauser et al., 2017, Marton et al., 2019).

A larger impairment for easier decisions in the current context is partly aligned with real-world behavior, where common OCD manifestations include uncertainty about simple perceptual events such as whether a lock is turned the correct way, a stove is off, or the individual caused physical harm to another person. On the surface this appears at odds with work suggesting that OCD is characterized by inflexible enhanced error monitoring (Endrass et al., 2010, Riesel et al., 2019). These studies found that the error-related negativity was larger in individuals with OCD when errors were less important, including when speed was emphasized over accuracy and when mistakes were not penalized. It would seem that inflexible monitoring should then also apply to easier decisions, where errors are not expected to occur, and perhaps result in increased rather than decreased performance. However, additional error *monitoring* does not necessarily translate into the successful use of additional information, and while monitoring may be enhanced, OCD symptoms appear to also be associated with impairments in at least some circumstances. Indeed, these complementary differences are suggestive of a different hypothesis: true (but potentially subtle) performance impairments may be overgeneralized and result in increased monitoring for errors across a variety of domains and circumstances, in turn also driving “not just right” experiences (Coles et al., 2003) and feelings of “incompleteness” (Summerfeldt et al., 2004). While such impairments appear not to always be accompanied by increased caution, reductions in drift rate can contribute to slower responding, and this may be perceived as a form of perseveration during real world behavior.

We found that changes in aggregate gamma and beta-band power were steeper for easier decisions, and moreover, the slope of aggregate power in each band was independently associated across subjects with drift rate measured based on behavioral data alone—for all conditions for the gamma band, and for five out of six conditions for the beta band. In line with expectation, the slope of aggregate beta-band power was negatively correlated with OCD score in five out of six conditions, with a trending effect in the remaining condition. However, the relationship between OCD score and drift rate could not be explained by this latter association.

Increases in gamma-band power were strongest at posterior and lateral electrode sites, while decreases in beta-band band power were strongest at parieto-occipital electrode sites. We also found smaller effects in the opposite direction in each band at more frontal electrodes. This contrasts with Polanía et al. (2014), where parietal electrodes were the source of positive ramping gamma-band effects during a perceptual decision task, and there were no electrodes showing negative changes. In addition, their study did not find a relationship between beta-band power and evidence integration during perceptual decision making, but reported decreases in beta-band power at frontal electrodes during an analogous reward-based task. It should be noted however that the perceptual task used by Polanía et al. (2014) differed from the current study, and involved making a size judgment based on a static stimulus instead of a motion judgment based on a dynamic stimulus. While overlapping, the two tasks also likely index different neural systems. Donner et al. (2009) used MEG for improved spatial resolution and a motion task very similar to the one used here, and found a priori defined localized signatures of evidence integration in both bands in premotor and motor cortex. MEG can likewise be used in the current context to separate beta-band activity originating from disparate neural sources. This would allow testing whether a more localized measure of beta-band power can better explain the relationship between drift rate and OCD symptoms compared to the more diffuse activity captured in the EEG.

Beta and gamma band power are also not the only candidate EEG-based neural mechanisms for explaining OCD-related evidence integration deficits. For example, multiple studies have shown that the centro-parietal positivity is related to evidence integration, as well as to estimates of drift rate based on behavioral data (O’Connell et al., 2012, van Vugt et al., 2019). It is also possible that EEG, and even MEG, are too coarse to tease out a neural signature of this relationship. At present, the search for a neural mechanism explaining these data is only loosely constrained. While the drift-diffusion model provides a formal description of deliberation, it operates at a level of abstraction that does not make detailed contact with neural mechanisms. Additional constraints may be derived through more biophysically realistic modeling of OCD-related perceptual deficits. For example, Wang and colleagues have investigated one class of models for solving the type of binary choice motion discrimination task used here which may be useful toward this end (Wang, 2002, Wong and Wang, 2006). Although the implementation details have differed between different instantiations, different versions have shared common important core features, including slow NMDA mediated recurrent excitation, properly tuned absolute and relative levels of excitation and inhibition throughout the decision, and a properly calibrated AMPA:NMDA receptor ratio. The structure and parameters of such models could be investigated to determine which changes could give rise to the pattern of data observed in relation to OCD symptoms. Similar features are also important for working memory maintenance, and a related model has been used to better understand working memory deficits in schizophrenia, which interestingly shares enhanced comorbidity with OCD (Buckley et al., 2009, Wang, 2006).

In summary, we replicated the link seen in prior work between OCD symptoms and computationally defined perceptual decision making deficits, and between gamma and beta-band power and perceptual evidence integration. Seeking to unify these disparate literatures, as predicted, we found a relationship between the slope of beta-band power and OCD symptoms. Although this relationship did not explain OCD-related drift rate impairments, our work provides additional constraints on the potential neural mechanisms underlying this deficit.

## Funding

This work was funded by the University of Maryland-College Park.

## Code

Source code for the models is available at: https://osf.io/kx9ry/.

## Declaration of Competing Interest

The authors declare that they have no known competing financial interests or personal relationships that could have appeared to influence the work reported in this paper.
