# Supplementary material for: The relationships between subclinical OCD symptoms, beta/gamma-band power, and the rate of evidence integration during perceptual decision making
Source: Neuroimage Clin. 2022 Feb 28;34:102975. doi: 10.1016/j.nicl.2022.102975 (PMC8904622; doi:10.1016/j.nicl.2022.102975)
Supplement: Supplementary data 1 [file mmc1.pdf]

Supplementary Figures

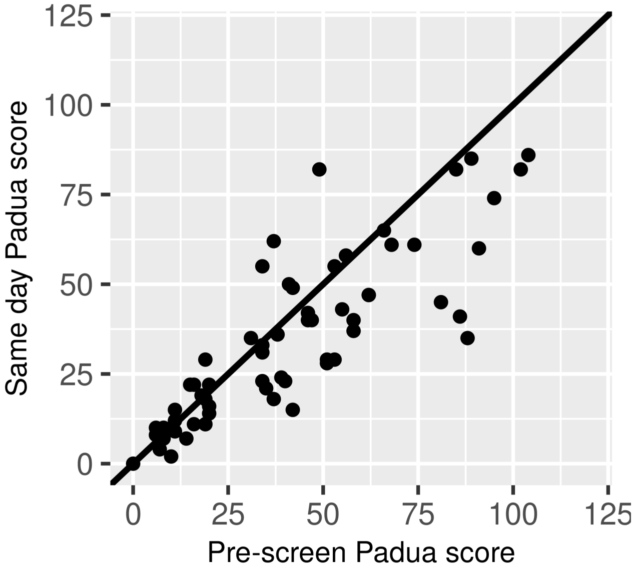

Figure S1: Same day versus pre-screen Padua Inventory scores. Line is the diagonal reference line (slope=1, intercept=0). Five participants from early in scheduling were not pre-screened and are excluded in this plot.

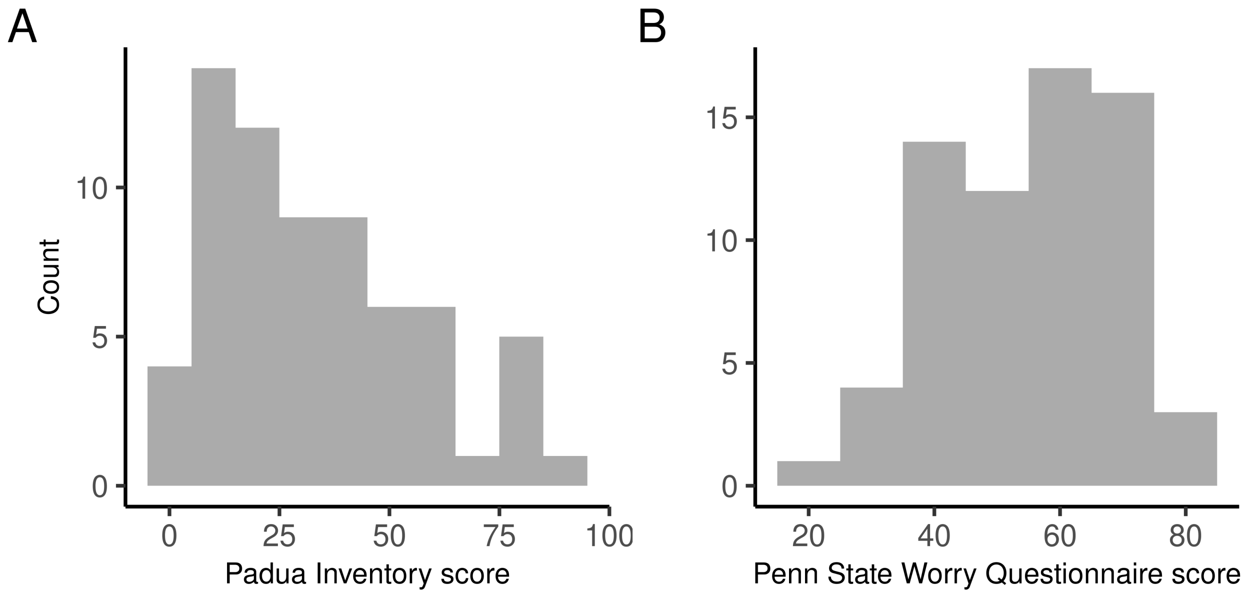

Figure S2: Histograms of same day Padua Inventory and Penn State Worry Questionnaire scores.

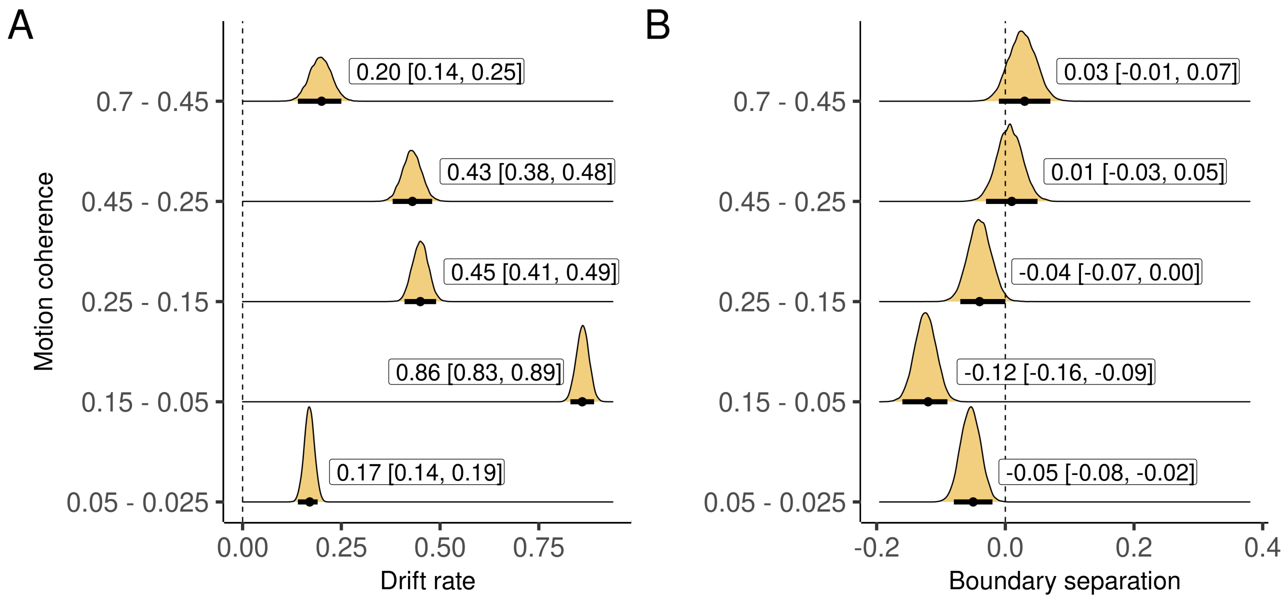

Figure S3: Differences between conditions for drift rate and boundary separation. Plots are marginal posterior distributions with medians and 95% credible intervals noted.

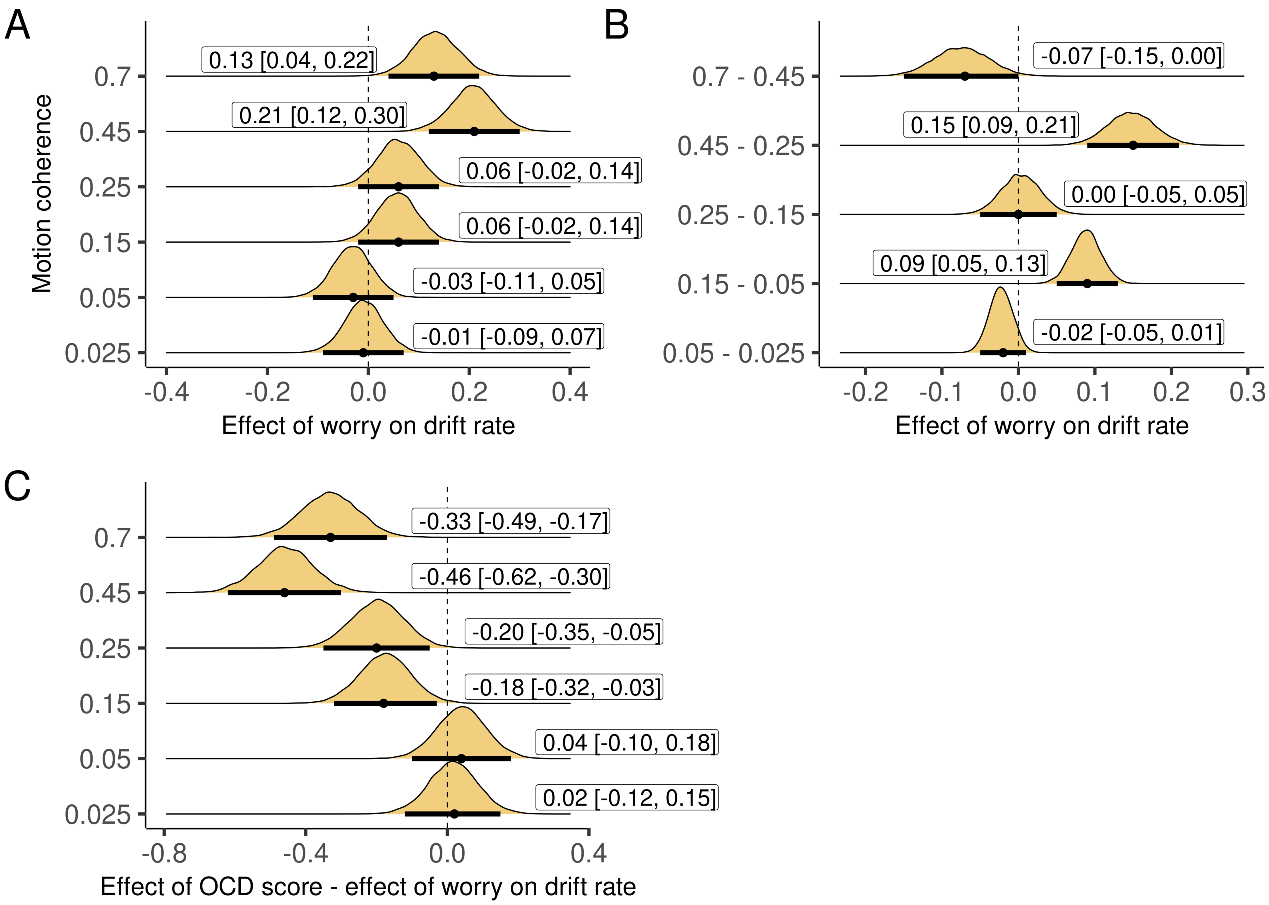

Figure S4: Effect of worry on drift rate, simultaneously controlling for OCD score. Plots are marginal posterior distributions with medians and 95% credible intervals noted.

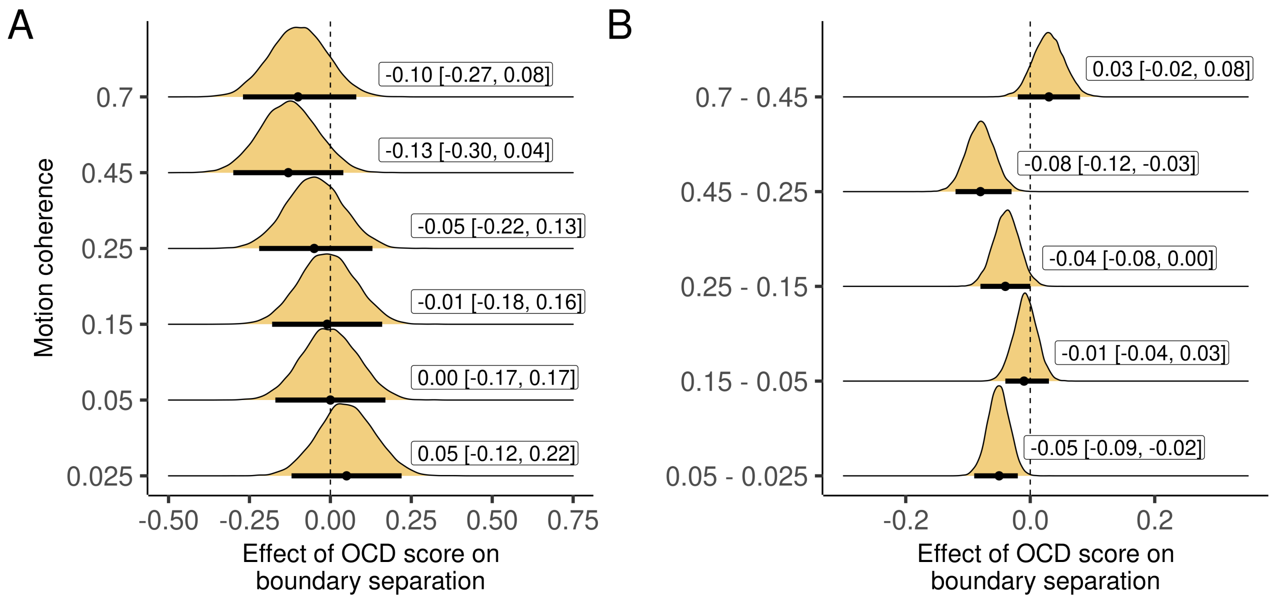

Figure S5: Effect of OCD score on boundary separation, simultaneously controlling for worry. Plots are marginal posterior distributions with medians and 95% credible intervals noted.

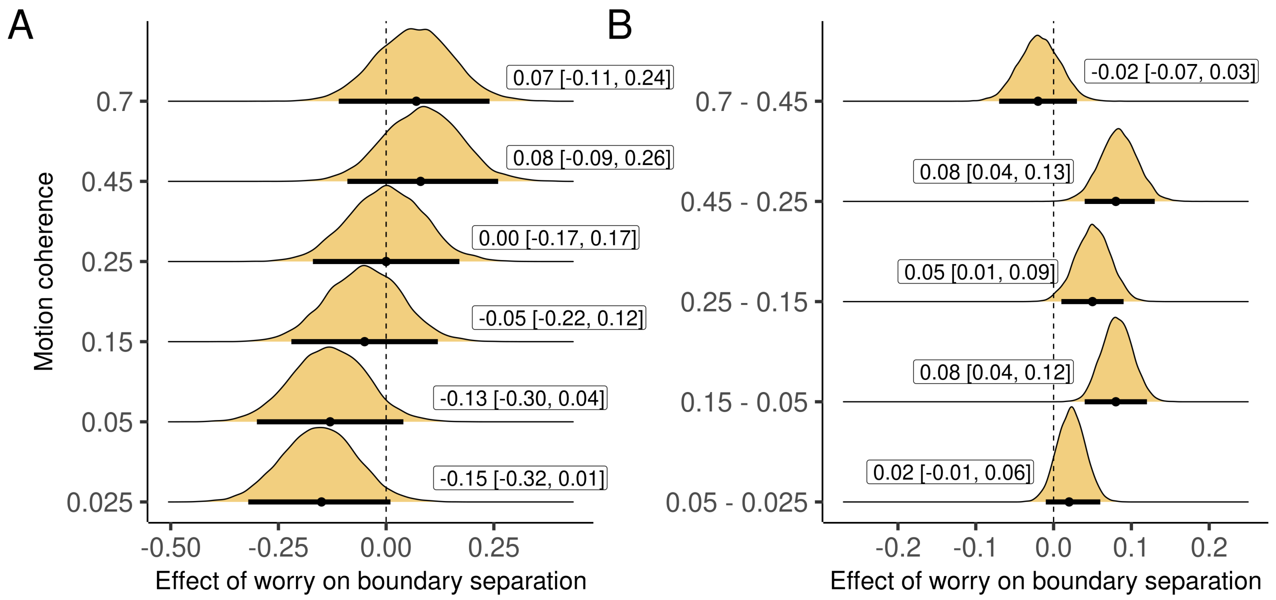

Figure S6: Effect of worry on boundary separation, simultaneously controlling for OCD score. Plots are marginal posterior distributions with medians and 95% credible intervals noted.

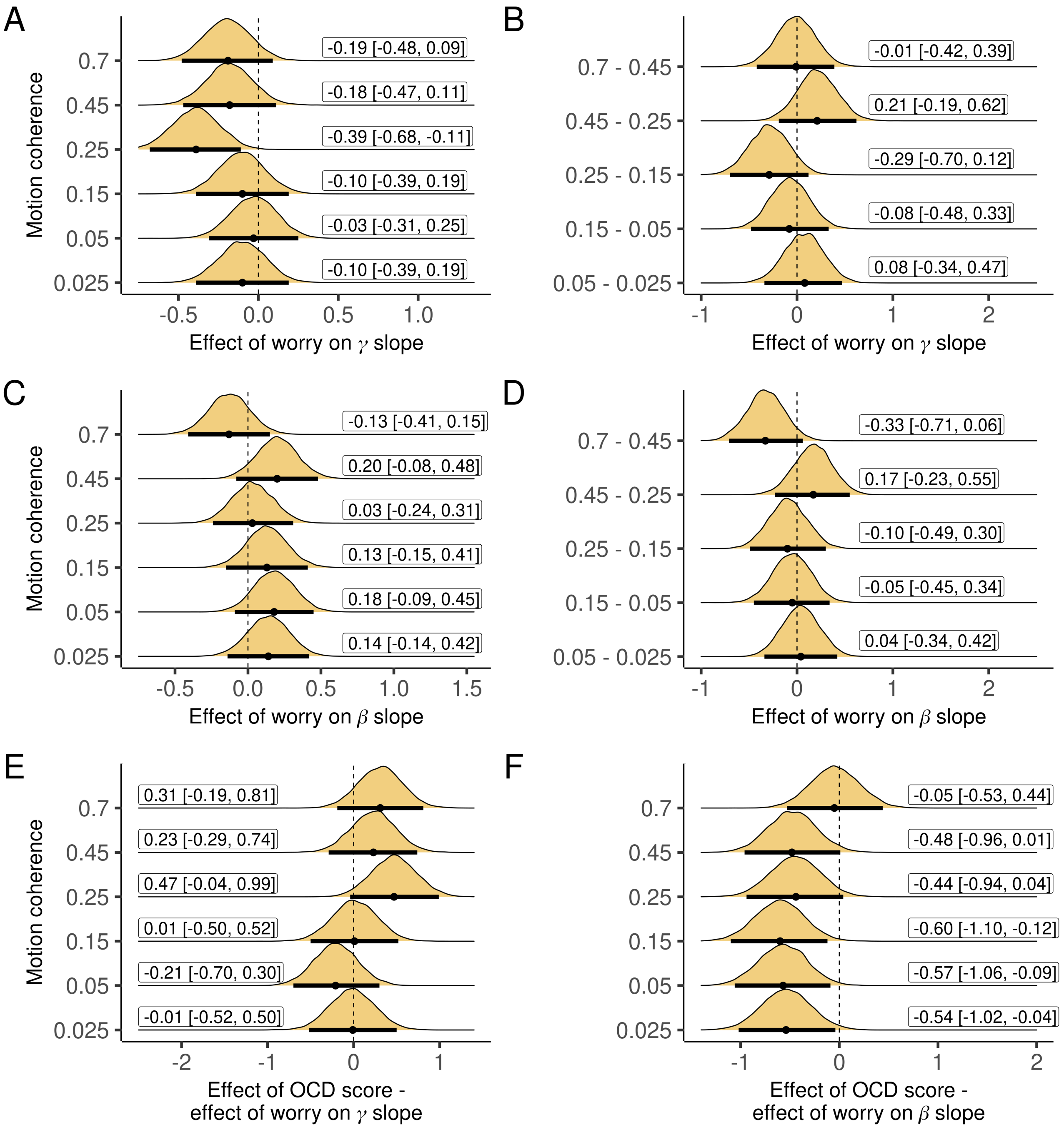

Figure S7: Effect of worry on the slope of whole brain aggregate gamma and beta-band power, across subjects. Plots are marginal posterior distributions with medians and 95% credible intervals noted.

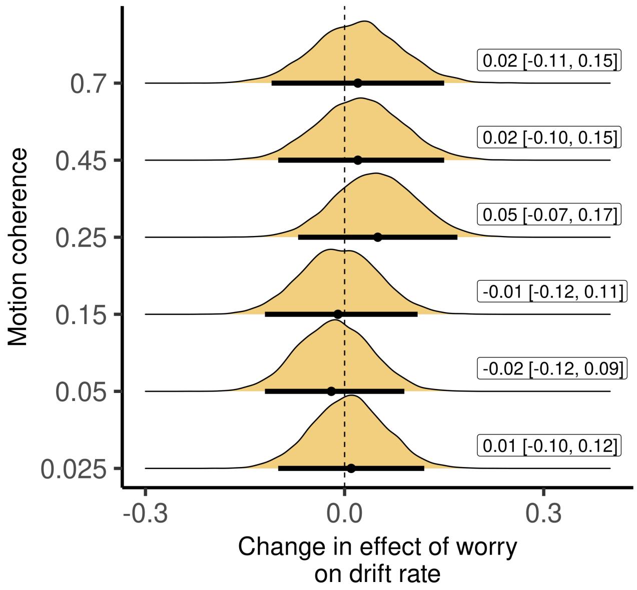

Figure S8: Change in the effect of worry on drift rate when controlling for the slopes of aggregate γ and β power in addition OCD score. Plots are marginal posterior distributions with medians and 95% credible intervals noted.
